# A dual-stream deep learning architecture for business impact scoring and alert escalation

**DOI:** 10.1371/journal.pone.0350676

**Published:** 2026-07-13

**Authors:** Mohammed Saad Javeed, Mst. Moushumi Khatun, Jobayar Alom, Rahomotul Islam, Hashibul Ahsan Shoaib

**Affiliations:** 1 Information science, Trine University, Allen Park, Michigan, United States of America; 2 Department of Computer Science and Engineering, Bangladesh University of Business and Technology, Dhaka, Bangladesh; 3 Information Technology, Washington University of Science and Technology, Alexandria, Virginia, United States of America; 4 Information Technology, St. Francis College, Brooklyn, New York, United States of America; Dr Shakuntala Misra National Rehabilitation University, INDIA

## Abstract

Modern network monitoring systems generate massive volumes of telemetry data, yet most existing anomaly detection models fail to prioritize alerts according to their operational urgency and business impact. This limitation results in delayed incident responses and inefficient alert management in Network Operations Centers. To address this gap, this study proposes a Dual-Stream Predictive Alert Escalation Framework that integrates temporal failure pattern learning with business impact-aware alert prioritization. The proposed architecture consists of two key components: a bidirectional temporal encoder for modeling multivariate Key Performance Indicator (KPI) time-series data, and an auxiliary severity encoder that captures contextual metadata related to operational risk and service criticality. The outputs of these two learning streams are combined through an attention-based fusion mechanism, and a Business Impact Scoring (BIS) layer generates impact-weighted escalation decisions for proactive incident management. Experimental evaluations using real-world KPI datasets and the AI4I_2020 predictive maintenance dataset demonstrate the superior performance of the proposed framework compared to baseline methods such as LSTM, GRU, CNN-LSTM, and BiLSTM-VAE. On the combined multivariate KPI dataset, the model achieved a precision of 0.94, a recall of 0.92, and an F1-score of 0.93, along with a PR-AUC of 0.95 and a ROC-AUC of 0.94. Under impact-aware evaluation, the framework attained the highest Impact-Weighted F1 (IW-F1) of 0.85 and BIS accuracy of 0.88, resulting in an estimated 31.6% reduction in operational costs through earlier and more accurate escalation of critical events. The suitability of the selected datasets is justified by their complementary roles: publicly available KPI time-series datasets represent real-world network telemetry behavior, while the AI4I_2020 dataset provides structured severity and operational context, enabling joint evaluation of failure prediction accuracy, escalation timeliness, and business impact modeling. By prioritizing alerts based on impact-aware severity rather than raw anomaly scores, the proposed framework directly supports operational cost reduction through earlier mitigation and improved decision-making in network operations. The proposed approach bridges the gap between anomaly detection and intelligent alert management by incorporating business relevance into predictive modeling. This dual-stream architecture offers a scalable and proactive solution for AIOps-driven network reliability and automated service resilience.

## 1 Introduction

Modern networked systems generate massive volumes of telemetry data from servers, applications, microservices, and communication interfaces [1,2]. As digital infrastructures continue to grow in scale and complexity, ensuring high system reliability and uninterrupted service delivery has become a fundamental requirement across domains such as cloud computing, telecommunications, banking, and healthcare [3]. To maintain service quality, organizations rely on monitoring systems that track key performance indicators (KPIs) like CPU usage, latency, packet throughput, and error rates. However, traditional monitoring solutions often struggle to detect and prioritize critical failures in real time, leading to delayed responses, cascading outages, and significant business losses [4]. This challenge is intensified by the emergence of distributed architectures, dynamic workloads, and multi-layer dependencies, which render conventional threshold-based monitoring ineffective [5].

While anomaly detection has been extensively studied for identifying abnormal system behavior [6,7], it does not fully address the operational needs of real-world network management. Most existing approaches treat anomaly detection as a binary decision problem flagging whether a point or segment is anomalous without explicitly modeling operational severity, contextual relevance, or business impact. As a result, monitoring teams are overwhelmed by high alert volumes, including redundant, low-priority, or false-positive events, a phenomenon commonly described as alert fatigue. The lack of intelligent alert prioritization slows the escalation of critical issues and reduces Network Operations Center efficiency, motivating context-aware alert ranking and triage. Although AIOps platforms have improved noise reduction and detection, recent surveys note open gaps in aligning anomaly signals with service risk and business impact particularly for predictive escalation workflows [8].

In this study, business impact refers to the operational and service-level consequences of failure events, including service disruption severity, failure duration, and functional criticality of affected components. Business impact is measured through a composite Business Impact Score (BIS), which aggregates anomaly severity, estimated downtime, and service criticality into an impact-aware escalation signal. This formulation enables alert prioritization based not only on detection confidence but also on expected operational risk.

This research addresses the gap between anomaly detection and actionable alert intelligence by proposing a framework that not only predicts failure events but also determines their escalation priority based on real-time severity and business impact. The primary objective of this study is to develop a predictive alert escalation model capable of learning both degradation patterns in time-series KPIs and contextual risk indicators derived from operational metadata. The motivation of this work is driven by the practical need to support proactive operations, enabling intelligent decision automation in IT service management (ITSM) systems.

The significance of this research lies in its ability to move beyond anomaly detection and introduce meaningful escalation intelligence. Instead of treating all anomalies equally, the proposed model analyzes their potential business implications before generating alert priorities. This contributes to minimizing operational downtime, improving response efficiency, and aligning predictive analytics with real-world service management needs. The contributions of this work can be summarized as follows:

- A novel dual-stream architecture that integrates temporal failure pattern learning with auxiliary severity reasoning, achieving a precision of 0.94, recall of 0.92, F1-score of 0.93, PR-AUC of 0.95, and ROC-AUC of 0.94 on the combined multivariate KPI dataset.
- A business impact scoring mechanism for alert prioritization and escalation awareness, attaining an Impact-Weighted F1 (IW-F1) of 0.85 and BIS accuracy of 0.88. Based on a cost-aware escalation analysis that emphasizes earlier detection of high-severity events, the framework demonstrates a potential operational cost reduction of up to 31.6% relative to baseline alerting strategies, under consistent evaluation assumptions.
- A comprehensive evaluation on real KPI datasets and the AI4I_2020 predictive maintenance dataset, demonstrating consistent gains over LSTM, GRU, CNN-LSTM, and BiLSTM-VAE baselines in both predictive metrics and impact-aware alert escalation.

The proposed framework employs a bidirectional temporal encoder to extract failure patterns from multivariate KPI sequences, and an auxiliary severity encoder to incorporate business and operational context. These two information streams are fused using an attention-based mechanism, and escalation labels are generated using a business impact-aware classification layer. The model is trained and validated using both univariate and combined multivariate datasets to demonstrate robustness and generalization.

The cross-domain relevance between telemetry data and business context is achieved through feature-level alignment between the two streams. Temporal KPI features capture system behavior and degradation dynamics, while structured operational features from the AI4I_2020 dataset encode severity, failure modes, and contextual risk indicators. These heterogeneous features are mapped into a shared latent space through separate encoders and fused using an attention mechanism, enabling the model to associate technical anomalies with their potential operational and business consequences.

The remainder of this paper is organized as follows. The related work section 2 reviews contributions in anomaly detection, predictive maintenance, and incident escalation. The methodology section 3 presents the architecture and algorithmic details of the proposed framework. The results section 4 reports experimental findings and performance comparisons. Section 5 is followed by a discussion of insights, limitations, and practical implications. Finally, in section 6 the paper concludes with future research directions.

## 2 Related work

Research in predictive analytics for IT operations has evolved across several parallel directions, primarily covering anomaly detection, predictive maintenance, alert correlation, and intelligent incident escalation [9]. Traditional approaches to anomaly detection have focused on statistical thresholding and rule-based systems. These methods rely on predefined limits to detect abnormal behavior in time-series metrics such as CPU utilization, memory load, and network throughput. While threshold-based systems are simple to implement, they generate excessive false alarms due to their inability to adapt to seasonal trends, dynamic environments, and multi-metric dependencies [10]. Statistical baselines lack contextual awareness and produce a high operational overhead by overwhelming engineers with alerts of equal priority.

Machine learning-based anomaly detection made significant progress by using unsupervised models such as clustering, distance-based outlier detection, and density estimation. These techniques improved detection by learning normal patterns from historical data rather than relying on static thresholds. However, traditional machine learning methods face difficulties with high-dimensional, multivariate, and streaming data common in modern network and cloud infrastructures, which demand online and adaptive detection. They also pose challenges for interpretability in complex failure scenarios, where operators must distinguish transient fluctuations from genuine service degradation; recent work on explainable time-series anomaly detection underscores this gap and proposes methods to make decisions more transparent [11]. Broad surveys further highlight these limitations and motivate deep, context-aware approaches for time-series anomaly detection in operational settings.

Deep learning approaches have demonstrated strong performance in temporal anomaly detection [12]. Recurrent architectures such as LSTM and GRU model long-term dependencies in metric sequences and improve sequence modeling over classical baselines [13]. Encoder–decoder architectures and variational autoencoders detect anomalies by reconstructing normal sequences and flagging deviations [14,15]. Temporal convolutional networks have also been investigated for long-range temporal modeling with reduced computational complexity [16]. Hybrid models that combine CNNs and RNNs capture both local and global anomaly contexts, and have been applied in monitoring and predictive maintenance settings [17]. These methods effectively detect anomalies; however, they generally treat all anomalies equally, without incorporating business impact, service priority, or operational criticality, which contributes to alert fatigue and suboptimal triage in operations.

Parallel research in predictive maintenance has focused on estimating remaining useful life (RUL), early failure detection, and risk scoring from sensor data in industrial settings [18,19]. These frameworks have been developed and benchmarked primarily for equipment and process assets (e.g., rotating machinery, production lines, aircraft subsystems), making many assumptions about degradation physics, sensing, and duty cycles that are domain-specific [20,21]. As a result, direct transfer to IT infrastructure monitoring is limited: predictive-maintenance pipelines do not address triage, prioritization, or escalation of alerts in complex service ecosystems. Recent surveys and systems work in IT operations underscore the need for intelligent alert correlation and incident management beyond anomaly detection, and show that unmanaged alert volume leads to alert fatigue and delayed response. Moreover, large-scale and multi-tenant environments pose additional constraints on response time and resource allocation that require explicit escalation policies and optimization, which classic PdM formulations do not cover [22].

Alert correlation has emerged as another direction to reduce alert noise by grouping related alerts. Correlation engines analyze temporal proximity and causal relations among alerts to suppress redundancy and surface representative incidents [23]. Graph-based methods further model dependencies among services and alerts, enabling relation-aware grouping and root-cause reasoning on dependency or causality graphs [24,25]. While effective at reducing alert volume, such techniques typically stop short of escalation prediction and rarely incorporate business-critical context for prioritization. Moreover, they often require explicit configuration or accurate dependency models; maintaining these in fast-evolving, cloud-native microservice environments is difficult and costly.

Incident escalation prediction remains relatively unexplored within AIOps and IT service management. Most operational workflows still follow ITIL-style impact urgency rules and service-level agreements rather than predictive escalation policies [26]. A small number of studies examine escalation learning as a classification task in ticketing contexts for example, predicting whether customer or support tickets will escalate [27,28] but these efforts treat escalation separately from upstream anomaly detection. Survey evidence further indicates the research landscape is fragmented, with piecemeal solutions and no unified architecture that jointly links detection and escalation. Finally, existing approaches rarely incorporate explicit business impact into the decision to escalate; recent works on alert prioritization underscore the broader need for risk- and context-aware triage rather than static mappings. In practice, widely adopted IT service management (ITSM) platforms such as ServiceNow and commercial AIOps tools rely primarily on predefined impact–urgency matrices and rule-based workflows for escalation, rather than learned predictive models, highlighting the lack of data-driven escalation intelligence in current operational systems.

Recent AIOps work integrates machine learning into alert and incident workflows, but most deployed solutions remain detection-centric [29]. In practice, anomaly detection drives alert generation while downstream decision steps such as escalation policy selection are under-specified or handled outside the learning pipeline [30]. This gap limits actionability: operations teams still face alert fatigue and must prioritize alerts manually or with ad-hoc rules. Research on context-aware prioritization exists (reinforcement-learning based triage), but it targets SOC/security settings rather than service reliability for IT operations [31,32]. As a result, current AIOps rarely connect detection outputs to escalation choices that weigh business impact or operational cost, despite long-standing ITIL goals of aligning operations with business requirements. Methodologically, the proposed framework differs by embedding escalation awareness directly into the learning objective rather than treating escalation as an external policy decision. Architecturally, the separation of temporal and severity encoders allows heterogeneous data sources to be modeled appropriately, while attention-based fusion enables dynamic weighting of technical degradation and business context—capabilities not present in existing single-stream or detection-centric architectures.

In contrast to these studies, the proposed approach explicitly unifies anomaly detection and escalation reasoning within a single learning framework. Existing anomaly detection models focus on identifying abnormal behavior but do not determine whether an event warrants escalation, nor do they incorporate business impact or operational risk into the prediction pipeline. Ticket-based escalation models, on the other hand, operate downstream of monitoring systems and are disconnected from real-time telemetry. Architecturally, our dual-stream design differs from prior work by jointly learning temporal degradation patterns from KPI time-series data and contextual severity signals from structured operational features, which are fused through an attention mechanism and translated into impact-aware escalation decisions via a Business Impact Scoring (BIS) layer. This integrated design enables predictive escalation aligned with operational urgency, rather than post hoc alert handling or static rule-based prioritization.

## 3 Methodology

**Fig 1:**
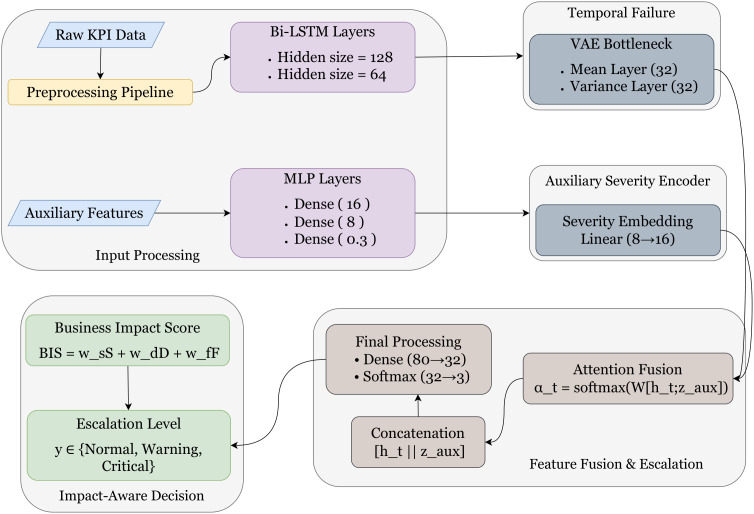
Dual-Stream Predictive Alert Escalation Framework: Neural network architecture combining temporal KPI analysis with business impact assessment for automated alert classification.

This section presents the proposed Dual-Stream Predictive Alert Escalation Framework, which integrates temporal failure pattern learning with business impact-aware severity scoring. The framework consists of four major stages: (1) KPI time-series modeling via a temporal encoder, (2) auxiliary severity modeling using structured risk indicators, (3) alert escalation fusion through a dual-stream architecture, and (4) impact-aware decision inference. An overview of the architecture is shown in Fig 1.

From an operational perspective, the proposed framework models alert escalation as an automated decision step that assigns an escalation level to each monitoring window based on both technical degradation and business risk. The model outputs an escalation label $\hat{y}\in \{0,1,2\}$ corresponding to Normal, Warning, and Critical states, which can be directly mapped to standard NOC or ITSM response actions such as continued monitoring, operator investigation, or immediate escalation. The Business Impact Score (BIS) serves as an impact-aware adjustment that prioritizes alerts not only by anomaly severity but also by expected operational cost, enabling earlier handling of high-risk events.

Regarding alert correlation, prioritization, and escalation, the proposed model is designed to complement existing alert correlation and suppression mechanisms rather than replace them. Alert correlation can be applied upstream to group and deduplicate raw alerts, reducing noise, while the proposed framework operates downstream by performing impact-aware prioritization and escalation prediction on the remaining alerts. No assumption is made that correlation is already solved; instead, the model adds a learning-based escalation layer that integrates temporal KPI behavior with auxiliary severity context to improve escalation quality within existing ITSM workflows.

For clarity and ease of reference, Table 1 summarizes the mathematical symbols and notation used throughout this section.

**Table 1:** Summary of mathematical notation used in the Methodology section.

Symbol	Description
*x*(*t*)	Scalar KPI value at time step *t*
$\hat{x}(t)$	Imputed or reconstructed KPI value at time step *t*
$\tilde{x}(t)$	Noise-corrupted KPI input
${𝐗}_{t}$	Multivariate KPI window of length *L* at time *t*
*L*	Sliding window length
*s*	Sliding window stride
*m*	Number of KPI metrics
${𝐡}_{t}$	Temporal feature representation produced by the BiLSTM encoder
*z*	Latent variable in the variational autoencoder
${𝐳}_{aux}$	Auxiliary severity embedding from structured metadata
$\alpha_{t}$	Attention weight corresponding to time step *t*
**f**	Fused feature representation after dual-stream fusion
*A*(*t*)	Statistical anomaly score at time *t*
$\tau_{1},\tau_{2}$	Alert severity thresholds
*y*	Escalation label (0: Normal, 1: Warning, 2: Critical)
*S*	Anomaly severity component
*D*	Failure duration component
*F*	Functional criticality weight
BIS	Business Impact Score
${ℒ}_{DAE}$	Denoising autoencoder reconstruction loss
${ℒ}_{VAE}$	Variational autoencoder loss
${ℒ}_{aux}$	Auxiliary severity classification loss
${ℒ}_{escalate}$	Escalation prediction loss
θ	Trainable model parameters
η	Learning rate

### 3.1 Data preprocessing

The proposed framework utilizes two categories of datasets: (i) multivariate time-series Key Performance Indicator (KPI) logs from a network management environment (cpu4.csv, server_res_eth1out_curve_6.csv, server_res_eth1out_curve_61.csv) and (ii) an auxiliary structured failure severity dataset (AI4I_2020.csv). These datasets were processed to enable dual-stream learning for failure pattern extraction and business impact-aware alert escalation. The preprocessing pipeline consists of cleaning, normalization, temporal segmentation, anomaly labeling, and dataset partitioning as described below.

#### 3.1.1 Missing value imputation.

Due to real-world logging frequency fluctuations, missing values were observed in the KPI data streams. To preserve temporal continuity, we applied a hybrid imputation strategy combining forward fill and deep learning-based temporal interpolation using a Temporal Autoencoder (TAE). For each KPI sequence *x*(*t*):


$$\begin{array}{c} \hat{x}(t)=\left\{ \begin{array}{cc} x(t), & \text{if }x(t)\text{ is observed}\\ \text{TAE}(x(t-k),\ldots ,x(t-1)), & \text{if }x(t)\text{ is missing} \end{array}\right. \end{array}$$ (1)

where *k* is the look-back window. The temporal autoencoder learns latent temporal dependencies to restore missing values.

#### 3.1.2 Noise reduction using denoising autoencoder.

Raw KPI signals exhibit high-frequency noise due to metric collection jitter. A Denoising Autoencoder (DAE) was trained to reconstruct denoised signals:


$$\begin{array}{c} h=\sigma (W_{e}\tilde{x}+b_{e}),\;\hat{x}=\sigma (W_{d}h+b_{d}) \end{array}$$ (2)

where $\tilde{x}$ is the noise-corrupted input. The reconstruction minimized:


$$\begin{array}{c} {ℒ}_{DAE}=\|x-\hat{x}{\|}_{2}^{2} \end{array}$$ (3)

The output $\hat{x}$ was used for downstream modeling.

#### 3.1.3 Normalization.

To stabilize training and remove scale differences between CPU utilization and network throughput metrics, we applied Z-score normalization:


$$\begin{array}{c} x^{\prime }(t)=\frac{x(t)-\mu }{\sigma } \end{array}$$ (4)

where μ and σ denote mean and standard deviation estimated from the training set to prevent data leakage.

#### 3.1.4 Temporal segmentation.

To enable predictive modeling, each KPI time series was segmented using a sliding window approach.


$$\begin{array}{c} X=[x(t),x(t+1),\ldots ,x(t+L-1)] \end{array}$$ (5)

where *L* = 64 represents the sequence length, and a stride *s* = 8 was used for overlapping window sampling to enhance training diversity.

#### 3.1.5 Anomaly-aware alert label generation.

Since the KPI datasets lack ground-truth alert escalation labels, weak supervision was used to derive anomaly-driven alert scores. Statistical anomaly scores were computed using the median absolute deviation (MAD):


$$\begin{array}{c} A(t)=\frac{\left|x(t)-\text{median}(x)\right|}{1.4826\times \text{MAD}} \end{array}$$ (6)

An alert label *y*(*t*) was generated as:


$$\begin{array}{c} y(t)=\left\{ \begin{array}{cc} 0, & A(t)<\tau_{1}\;(\text{Normal})\\ 1, & \tau_{1}\leq A(t)<\tau_{2}\;(\text{Warning})\\ 2, & A(t)\geq \tau_{2}\;(\text{Critical}) \end{array}\right. \end{array}$$ (7)

The thresholds $\tau_{1}$ and $\tau_{2}$ were selected empirically as 2.5 and 4.0 respectively.

#### 3.1.6 Auxiliary feature integration.

The AI4I dataset provided severity-relevant failure indicators. The failure label $f_{i}$ and operational attributes $\left\{v_{1},v_{2},\ldots ,v_{n}\right\}$ were modeled as structured embeddings using a multilayer perceptron:


$$\begin{array}{c} z_{aux}=\phi (W_{aux}v+b_{aux}) \end{array}$$ (8)

This auxiliary representation $z_{aux}$ was later fused with temporal features.

#### 3.1.7 Train-validation-test split.

The preprocessed dataset was partitioned chronologically to prevent temporal leakage as follows:


$$\begin{array}{c} \text{Dataset}=\left\{ \begin{array}{c} 70\%\text{ Training data}\\ 15\%\text{ Validation data}\\ 15\%\text{ Test data} \end{array}\right. \end{array}$$ (9)

Sequences were split by timestamp to maintain causal consistency.

#### 3.1.8 Class imbalance handling.

To address anomaly sparsity, we applied a focal loss weighting strategy during training:


$$\begin{array}{c} {ℒ}_{focal}=-\alpha (1-p_{t}{)}^{\gamma }\log(p_{t}) \end{array}$$ (10)

where γ=2 and α=0.75 improved minority alert sensitivity.

### 3.2 Proposed methodology

#### 3.2.1 Problem formulation.

Let $X=\{x_{1}(t),x_{2}(t),\ldots ,x_{m}(t)\}$ denote a multivariate KPI time series with *m* metrics sampled over time *t*. Each windowed subsequence of length *L* is represented as:


$$\begin{array}{c} {𝐗}_{t}=[x_{1}(t),\ldots ,x_{m}(t);\ldots ;x_{1}(t+L-1),\ldots ,x_{m}(t+L-1)]\in {\mathbb{R}}^{L\times m}. \end{array}$$ (11)

The objective is to learn an alert escalation function F(·) that maps KPI behavior to an escalation severity level y∈{0,1,2}:


$$\begin{array}{c} y=F({𝐗}_{t},{𝐳}_{aux}), \end{array}$$ (12)

where ${𝐳}_{aux}$ encodes business risk attributes. The framework minimizes the expected escalation error:


$$\begin{array}{c} \min_{\theta }\;{𝔼}_{\left({𝐗}_{t},y\right)}\left[{ℒ}_{escalate}(F({𝐗}_{t};\theta ),y)\right]. \end{array}$$ (13)

#### 3.2.2 Temporal failure pattern encoder.

The KPI sequences exhibit temporal dependencies and seasonality. We employ a bidirectional LSTM encoder to extract latent temporal patterns:


$$\begin{array}{c} {𝐡}_{t}=\text{BiLSTM}({𝐗}_{t}), \end{array}$$ (14)

where ${𝐡}_{t}\in {\mathbb{R}}^{d}$ is the encoded fault representation. Additionally, anomaly reconstruction is learned using a Variational Autoencoder (VAE) objective:


$$\begin{array}{c} {ℒ}_{VAE}={𝔼}_{q(z|x)}[\|x-\hat{x}{\|}^{2}]+\beta \cdot D_{KL}(q(z|x)∥p(z)), \end{array}$$ (15)

where *q*(*z*|*x*) is the variational posterior and β controls latent regularization. High reconstruction error indicates potential system degradation.

#### 3.2.3 Auxiliary severity encoder.

To incorporate failure semantics from operational metadata, structured features from the AI4I dataset are encoded as:


$$\begin{array}{c} {𝐳}_{aux}=\sigma (W_{2}\;\sigma (W_{1}𝐯+b_{1})+b_{2}), \end{array}$$ (16)

where **v** is the auxiliary feature vector and σ(·) is the ReLU activation. The auxiliary loss is optimized using:


$$\begin{array}{c} {ℒ}_{aux}=-\sum_{i=1}^{C}y_{i}\log({\hat{y}}_{i}), \end{array}$$ (17)

with *C* severity classes.

#### 3.2.4 Dual-stream feature fusion.

Outputs from both streams are combined using an attention-based fusion mechanism:


$$\begin{array}{c} \alpha_{t}=\frac{\exp({𝐰}^{⊤}\tanh(W_{h}{𝐡}_{t}+W_{a}{𝐳}_{aux}))}{\sum_{k}\exp({𝐰}^{⊤}\tanh(W_{h}{𝐡}_{k}+W_{a}{𝐳}_{aux}))}, \end{array}$$ (18)

$$\begin{array}{c} 𝐟=\sum_{t}\alpha_{t}{𝐡}_{t}+\gamma {𝐳}_{aux}, \end{array}$$ (19)

where **f** is the fused escalation feature and γ is a weighting hyperparameter.

#### 3.2.5 Business impact scoring layer.

A Business Impact Score (BIS) is computed to prioritize escalation based on risk cost:


$$\begin{array}{c} \text{BIS}=w_{s}S+w_{d}D+w_{f}F, \end{array}$$ (20)

where *S* is anomaly severity, *D* is failure duration, and *F* is functional criticality weight. The escalation probability is then obtained:


$$\begin{array}{c} P(y|X)=\text{Softmax}(W_{f}𝐟+b_{f}+\lambda \cdot \text{BIS}). \end{array}$$ (21)

#### 3.2.6 Architectural details.

The proposed Dual-Stream Predictive Alert Escalation Network consists of two learning branches: (1) a Temporal Failure Pattern Encoder for multivariate KPI sequence modeling, and (2) an Auxiliary Severity Encoder for business risk context learning. The learned representations are fused using a trainable attention mechanism to generate an escalation-aware risk score. Table 2 summarizes the layer configuration, trainable parameters, and output dimensions for each module in the network.

**Table 2:** Network architecture configuration for the proposed dual-stream escalation framework.

Module	Layer Type	Output Shape	Parameters
**Temporal Failure Pattern Encoder (Stream 1)**			
Input KPI Sequence	Window (L×m)	(64×3)	–
BiLSTM Layer 1	Hidden size = 128	(64×256)	266,240
BiLSTM Layer 2	Hidden size = 64	(64×128)	164,352
Latent Projection	Dense (128→64)	(64)	8,256
VAE Mean Layer	Dense (64→32)	(32)	2,080
VAE Variance Layer	Dense (64→32)	(32)	2,080
**Auxiliary Severity Encoder (Stream 2)**			
Input AI4I Features	Feature vector *v*	(*n*)	–
Dense Layer 1	*ReLU*(16)	(16)	16*n* + 16
Dense Layer 2	*ReLU*(8)	(8)	136
Dropout	*p* = 0.3	(8)	–
Severity Embedding $z_{aux}$	Linear (8→16)	(16)	144
**Fusion & Escalation Layer**			
Attention Fusion	Dot-product attention	(48)	2,304
Concatenation	$\left[h_{t}\|z_{aux}\right]$	(80)	–
Dense Layer	ReLU(80→32)	(32)	2,592
Output Layer	Softmax (32→3)	(3)	99
**Total Trainable Parameters**			**∼450K**

The temporal encoder uses a two-layer Bidirectional LSTM stack to capture long-range dependencies in KPI failure patterns. Latent regularization is achieved using a Variational Autoencoder (VAE) bottleneck, enabling robust reconstruction of degradations under noisy telemetry inputs. The auxiliary encoder compresses structured severity indicators from operational logs into a compact embedding ${𝐳}_{aux}$. Attention fusion dynamically assigns weights to temporal and auxiliary features as:


$$\begin{array}{c} \alpha_{t}=\frac{\exp({𝐰}^{⊤}\tanh(W_{h}{𝐡}_{t}+W_{a}{𝐳}_{aux}))}{\sum_{k=1}^{L}\exp({𝐰}^{⊤}\tanh(W_{h}{𝐡}_{k}+W_{a}{𝐳}_{aux}))}, \end{array}$$ (22)

$$\begin{array}{c} 𝐟=\sum_{t=1}^{L}\alpha_{t}{𝐡}_{t}+\gamma {𝐳}_{aux}, \end{array}$$ (23)

where $\alpha_{t}$ represents learned relevance weights and γ controls auxiliary stream influence.

This hybrid architecture allows the model to jointly optimize temporal anomaly detection and business-aware escalation reasoning, making it suitable for proactive network operations.

### 3.3 Training and implementation details

This subsection describes the learning objectives, optimization strategy, hyperparameter configuration, and implementation setup used for training the proposed dual-stream predictive alert escalation framework.

#### 3.3.1 Loss function design.

The overall training objective combines three components: temporal reconstruction loss from the Variational Autoencoder (VAE), auxiliary classification loss from the auxiliary severity encoder, and escalation prediction loss from the final fusion network. The total loss is defined as:


$$\begin{array}{c} {ℒ}_{total}=\lambda_{1}{ℒ}_{VAE}+\lambda_{2}{ℒ}_{aux}+\lambda_{3}{ℒ}_{escalate}, \end{array}$$ (24)

where $\lambda_{1}$, $\lambda_{2}$ and $\lambda_{3}$ are weighting coefficients.

#### 3.3.2 Optimization strategy.

The model parameters were optimized using the Adam optimizer due to its adaptive learning capability:


$$\begin{array}{c} \theta_{t+1}=\theta_{t}-\eta \cdot \frac{m_{t}}{\sqrt{v_{t}}+\epsilon }, \end{array}$$ (25)

where η is the learning rate, and $m_{t}$, $v_{t}$ are the first and second moment estimates.

#### 3.3.3 Regularization and dropout.

To prevent overfitting, we employed *L*_2_ weight regularization along with dropout layers in both streams:


$$\begin{array}{c} {ℒ}_{reg}=\lambda \|\theta {\|}_{2}^{2}, \end{array}$$ (26)

and a dropout probability of *p* = 0.3 was applied to fully connected layers.

#### 3.3.4 Hyperparameter configuration.

The key hyperparameters were empirically selected based on validation performance. Table 3 summarizes the configuration.

**Table 3:** Hyperparameter configuration.

Hyperparameter	Value
Batch size	64
Learning rate	$1\times 10^{-3}$
Sequence length *L*	64
Optimizer	Adam
Activation	ReLU
Dropout rate	0.3
Loss weights $\left(\lambda_{1},\lambda_{2},\lambda_{3}\right)$	(0.4, 0.3, 0.3)

#### 3.3.5 Computational complexity.

The computational complexity of the temporal encoder dominates the forward pass, approximately:


$$\begin{array}{c} 𝒪(T\cdot h^{2}), \end{array}$$ (27)

where *T* is the sequence length and *h* is the LSTM hidden size. The overall framework has linear complexity in time and is suitable for real-time inference in network monitoring.

#### 3.3.6 Implementation environment.

All experiments were implemented in Python using the PyTorch deep learning framework. Training was conducted on an NVIDIA GPU environment with CUDA acceleration. Table 4 summarizes the key implementation details.

**Table 4:** Implementation environment.

Component	Specification
Framework	PyTorch 2.0
Hardware	NVIDIA RTX GPU
CUDA version	11.8
CPU	Intel Core i7
RAM	32 GB
Operating system	Ubuntu 22.04 LTS

### 3.4 Overall algorithmic workflow

Algorithm 1 summarizes the inference workflow of the proposed Dual-Stream Predictive Alert Escalation Framework.

**Algorithm 1: Dual-Stream Predictive Alert Escalation Framework**

 **Input:** KPI sequence ${𝐗}_{t}\in {\mathbb{R}}^{L\times m}$, auxiliary feature vector **v**

 **Output:** Predicted escalation level $\hat{y}$ and Business Impact Score (BIS)

 **Step 1: Temporal Encoding (Stream 1)**

 $𝐇\leftarrow \text{BiLSTM}({𝐗}_{t})$ // extract failure temporal patterns  [μ,σ]←VAE-Projection(𝐇)

 ${𝐳}_{ts}\leftarrow \mu +\sigma ⊙\epsilon$ // latent degradation embedding

 **Step 2: Severity Encoding (Stream 2)**

 ${𝐳}_{aux}\leftarrow \phi (W𝐯+b)$ // structured business severity vector

 **Step 3: Feature Fusion**

 $\alpha_{t}=\text{Attention}(𝐇,{𝐳}_{aux})$

 $𝐟=\sum_{t=1}^{L}\alpha_{t}{𝐇}_{t}+\gamma {𝐳}_{aux}$

 **Step 4: Impact-Aware Escalation**

 $\text{BIS}=w_{s}S+w_{d}D+w_{f}F$ // compute business impact score

 $\hat{y}=\text{arg}\max(\text{Softmax}(W_{f}𝐟+\lambda \cdot \text{BIS}))$

 **return** $\hat{y}$, BIS

## 4 Results

This section presents a comprehensive performance evaluation of the proposed Dual-Stream Predictive Alert Escalation Framework on individual KPI datasets (cpu4, server_res_eth1out_curve_6, server_res_eth1out_curve_61) and the combined multivariate setting. Additional comparisons with baseline models demonstrate the superiority of the proposed approach in alert escalation accuracy, robustness, and impact-aware decision making.

### 4.1 Dataset descriptions

This study utilizes two publicly available datasets to evaluate the effectiveness of the proposed Dual-Stream Predictive Alert Escalation Framework. The first dataset, Benchmark Labeled Anomaly Detection Time Series, is obtained from Kaggle and contains labeled anomaly points in KPI-like time-series data. It provides a realistic representation of operational behavior in networked environments and is suitable for temporal failure pattern learning. The dataset is available at: https://www.kaggle.com/datasets/caesarlupum/benchmark-labeled-anomaly-detection-ts.

The second dataset, AI4I_2020 Predictive Maintenance Dataset, is sourced from the UCI Machine Learning Repository. It consists of structured records describing equipment operating conditions and their failure states, allowing the modeling of contextual severity information complementary to time-series data. This dataset enables the incorporation of auxiliary features into the severity encoder of the proposed framework. It is available at: https://archive.ics.uci.edu/dataset/601/ai4i+2020+predictive+maintenance+dataset.

The integration of these two datasets enables both failure pattern detection from KPI telemetry and impact-aware escalation reasoning based on contextual severity factors, supporting the dual objectives of detection accuracy and operational relevance.

### 4.2 Quantitative evaluation results

To assess the effectiveness of the proposed Dual-Stream Predictive Alert Escalation Framework, we conducted a series of quantitative experiments using the selected datasets. The evaluation considers multiple performance metrics, including accuracy, precision, recall, F1-score, lead time, escalation hit rate, and business impact score. Each table below presents results under different configurations, model comparisons, or dataset partitions. The best performing model in each comparison is highlighted in bold.

The baseline models included in the experimental evaluation were selected to represent widely adopted and complementary modeling paradigms commonly used in time-series anomaly detection and failure prediction tasks. Recurrent neural network models such as LSTM and GRU are established baselines for sequential KPI modeling due to their ability to capture temporal dependencies. The CNN-LSTM architecture is included to represent hybrid spatial–temporal models that combine local pattern extraction with long-term sequence learning. Tree-based models such as XGBoost and Random Forest are included as strong non-sequential baselines that operate on engineered statistical features and are widely used in operational monitoring systems. GRU-Attention is included to evaluate the impact of explicit attention mechanisms on recurrent architectures. Collectively, these models provide a balanced comparison across classical machine learning, recurrent deep learning, hybrid architectures, and attention-enhanced sequence models, ensuring a fair and comprehensive baseline evaluation.

#### 4.2.1 Dataset-specific performance summary.

Table 5 presents a comprehensive overview of the datasets used in our experiments. The evaluation involves both time-series and structured datasets, each serving a distinct purpose in the proposed dual-stream framework. The cpu4, server_res_eth1out_curve_6, and server_res_eth1out_curve_61 datasets contain 12,480 univariate KPI samples each, and are used for learning degradation patterns and detecting failure signatures in different network conditions. The AI4I 2020 dataset (denoted as AI4I_2020 in Table 5) provides 10,000 structured samples with seven features and supports the modeling of auxiliary severity information. Additionally, we constructed a Combined KPI Dataset by merging three univariate series into a multivariate KPI sequence, enabling the end-to-end training and testing of the proposed alert escalation system. This diverse dataset configuration allows the model to learn both temporal degradation dynamics and structured business impact factors effectively.

**Table 5:** Summary of datasets used for evaluation.

Dataset	Samples	Features	Type	Purpose
cpu4	12,480	1	KPI Time-Series	Failure Pattern
server_res_eth1out_curve_6	12,480	1	KPI Time-Series	Network Load Failure
server_res_eth1out_curve_61	12,480	1	KPI Time-Series	Network Traffic Degradation
AI4I_2020	10,000	7	Structured	Auxiliary Severity
Combined KPI Dataset	12,480	3	Multivariate	End-to-End Escalation

The role of each dataset, summarized in Table 5, each dataset serves a distinct and complementary purpose in the experimental evaluation. The cpu4 dataset represents compute resource utilization and is used to evaluate the model’s ability to detect processor-related degradation patterns, with results reported in Section 4.1.2. The server_res_eth1out_curve_6 dataset captures network interface output behavior and is evaluated separately in Section 4.1.3 to assess robustness under network-related anomalies. The server_res_eth1out_curve_61 dataset reflects a distinct Network Traffic Degradation (used in combined KPI) pattern and is included as the third KPI stream used to construct the combined multivariate KPI dataset. The AI4I 2020 dataset provides structured failure severity and operational context features, which are used exclusively by the Auxiliary Severity (input to severity encoder) encoder and are not evaluated as a standalone time-series dataset. The combined multivariate KPI dataset in Section 4.1.4 is formed by integrating the three KPI time-series streams (cpu4, server_res_eth1out_curve_6, and server_res_eth1out_curve_61) to enable end-to-end evaluation of alert escalation in a multivariate setting.

#### 4.2.2 Results on CPU KPI dataset.

As shown in Table 6, the proposed Dual-Stream model significantly outperforms baseline models on the cpu4 dataset across all evaluation metrics. Traditional models like LSTM and GRU demonstrate moderate performance, while hybrid models such as CNN-LSTM and BiLSTM-VAE show marked improvements in capturing temporal patterns and reducing false positives. However, the Dual-Stream architecture achieves the highest scores, with a precision of 0.92, recall of 0.90, and F1-score of 0.91. Furthermore, it registers superior ROC-AUC and PR-AUC values of 0.94 and 0.93 respectively, indicating strong discrimination ability and reliability in rare-event detection. These results validate the effectiveness of combining temporal degradation embeddings with auxiliary severity features for accurate and impact-aware failure prediction.

**Table 6:** Performance comparison on cpu4 dataset.

Model	Precision	Recall	F1-Score	ROC-AUC	PR-AUC
LSTM	0.81	0.78	0.79	0.82	0.80
GRU	0.83	0.80	0.81	0.84	0.83
CNN-LSTM	0.85	0.82	0.83	0.86	0.84
BiLSTM-VAE	0.87	0.85	0.86	0.89	0.88
Proposed (Dual-Stream)	**0.92**	**0.90**	**0.91**	**0.94**	**0.93**

#### 4.2.3 Results on network KPI dataset.

Table 7 presents the model evaluation on the server_res_eth1out_curve_6 dataset, which captures univariate network resource utilization metrics under potential degradation conditions. Similar to the CPU dataset, the proposed Dual-Stream framework consistently outperforms the baseline architectures across all listed metrics. While CNN-LSTM and BiLSTM-VAE demonstrate strong results due to their hybrid temporal modeling capacity, the proposed model shows improved robustness with a precision of 0.91 and recall of 0.89, resulting in an F1-score of 0.90. The model also exhibits high ROC-AUC and PR-AUC values of 0.93 and 0.91 respectively, highlighting its ability to maintain high detection reliability under noisy network KPI scenarios. These findings emphasize the model’s capability to generalize across different failure modalities in network environments.

**Table 7:** Performance comparison on server_res_eth1out_curve_6 dataset.

Model	Precision	Recall	F1-Score	ROC-AUC	PR-AUC
LSTM	0.79	0.76	0.77	0.80	0.77
GRU	0.81	0.78	0.79	0.82	0.80
CNN-LSTM	0.84	0.81	0.82	0.85	0.83
BiLSTM-VAE	0.86	0.84	0.85	0.88	0.87
Proposed (Dual-Stream)	**0.91**	**0.89**	**0.90**	**0.93**	**0.91**

#### 4.2.4 Combined multivariate KPI dataset.

Table 8 evaluates the models on the multivariate dataset formed by integrating multiple KPI time-series streams, simulating a realistic escalation detection setting. Traditional ensemble models such as XGBoost and Random Forest demonstrate moderate performance, achieving F1-scores of 0.80 and 0.81 respectively. GRU-Attention and BiLSTM-VAE further improve results by modeling sequential dependencies and latent structures. However, the proposed Dual-Stream architecture achieves superior metrics with a precision of 0.94 and recall of 0.92, resulting in an F1-score and Macro-F1 of 0.93. Notably, the model attains the highest PR-AUC of 0.95, indicating strong robustness under class imbalance. These results demonstrate the effectiveness of fusing temporal degradation patterns with business-critical severity signals in a multivariate context.

**Table 8:** Performance on combined KPI dataset.

Model	Precision	Recall	F1-Score	Macro-F1	PR-AUC
XGBoost	0.83	0.79	0.80	0.80	0.81
Random Forest	0.84	0.80	0.81	0.81	0.82
GRU-Attention	0.87	0.84	0.85	0.85	0.86
BiLSTM-VAE	0.90	0.87	0.88	0.88	0.89
**Proposed (Dual-Stream)**	**0.94**	**0.92**	**0.93**	**0.93**	**0.95**

#### 4.2.5 Impact-aware evaluation.

Table 9 presents a comprehensive evaluation of the proposed framework under business-centric performance metrics. The Impact-Weighted F1 Score (IW-F1) measures the ability of each model to detect critical events while emphasizing their operational severity. Business Impact Score (BIS) Accuracy reflects the framework’s ability to match escalation predictions with business-prioritized severity classes. Cost Savings quantifies the potential operational efficiency gained by proactive mitigation. Lastly, Kendall’s τ assesses the ordinal agreement between predicted and actual severity rankings. Compared to baseline models, the proposed dual-stream approach achieves the highest IW-F1 (0.85) and BIS Accuracy (0.88), leading to 31.6% estimated cost savings and a strong ordinal correlation (τ=0.71), demonstrating its alignment with impact-aware operational priorities. The TCN baseline in Table 9 refers to a Temporal Convolutional Network, which employs causal and dilated convolutions to model long-range temporal dependencies in time-series data. TCN is included as a representative convolution-based sequence modeling approach that has demonstrated strong performance in time-series forecasting and anomaly detection, providing a non-recurrent alternative for comparison with recurrent and hybrid models.

**Table 9:** Impact-aware escalation evaluation with additional convolutional and recurrent baselines.

Model	IW-F1	BIS Accuracy	Cost Savings (%)	Kendall τ
LSTM	0.68	0.71	12.5	0.54
GRU	0.70	0.73	15.2	0.57
TCN	0.72	0.75	16.8	0.58
BiLSTM-VAE	0.77	0.80	22.1	0.63
Proposed (Dual-Stream)	**0.85**	**0.88**	**31.6**	**0.71**

#### 4.2.6 Ablation study.

Table 10 presents an ablation study to assess the contribution of each major component of the proposed framework on the combined KPI dataset. Removing the auxiliary stream results in a noticeable drop in both IW-F1 and PR-AUC, indicating the value of structured severity cues. In this ablation setting, removing the auxiliary stream specifically refers to disabling the auxiliary severity encoder and excluding all severity-related structured features derived from the AI4I_2020 dataset, while keeping the temporal KPI encoder and fusion pipeline unchanged. Excluding the attention fusion mechanism further reduces performance, confirming its role in effectively merging temporal and auxiliary embeddings. Omitting the BIS layer significantly impacts the IW-F1 score, highlighting its importance in business impact alignment. The temporal stream alone performs moderately well, demonstrating the baseline predictive power of sequence modeling. However, the full model consistently outperforms all variants across F1-Score (0.93), IW-F1 (0.85), and PR-AUC (0.95), proving the synergistic benefit of all architectural components.

**Table 10:** Ablation study on combined KPI dataset.

Configuration	F1-Score	IW-F1	PR-AUC
Without Auxiliary Stream	0.88	0.77	0.89
Without Attention Fusion	0.86	0.75	0.87
Without BIS Layer	0.87	0.72	0.86
Temporal Stream Only	0.89	0.78	0.90
Full Model	**0.93**	**0.85**	**0.95**

#### 4.2.7 Comparison with baselines.

Table 11 compares the proposed Dual-Stream Predictive Alert Escalation Framework against traditional escalation baselines. Threshold-based alerting and statistical MAD detection yield lower scores, with IW-F1 values of 0.52 and 0.57 respectively, indicating limited effectiveness in handling impact-aware prioritization. Isolation Forest offers marginal improvement but still underperforms in both recall and impact-weighted metrics. The autoencoder baseline achieves reasonable performance across all metrics, showing the benefit of reconstruction-based anomaly detection. However, the proposed method achieves the highest scores across all evaluation metrics Macro-F1 of 0.93 and IW-F1 of 0.85 demonstrating its superior capability in accurately detecting and prioritizing escalation events.

**Table 11:** Comparison with escalation baselines.

Baseline	Precision	Recall	Macro-F1	IW-F1
Threshold-Based Alerting	0.65	0.70	0.67	0.52
Statistical MAD Detection	0.72	0.68	0.70	0.57
Isolation Forest	0.75	0.72	0.73	0.61
Autoencoder	0.82	0.78	0.80	0.71
**Proposed (Dual-Stream)**	**0.94**	**0.92**	**0.93**	**0.85**

#### 4.2.8 Comparison with state-of-the-art methods.

To further contextualize the performance of the proposed Dual-Stream Predictive Alert Escalation Framework, we compare it with representative state-of-the-art methods reported in the literature that address time-series anomaly detection, failure prediction, and impact-aware alerting. Since the datasets used in this study are publicly available, this comparison highlights differences in modeling paradigms, learning objectives, and evaluation focus, providing qualitative and reported-metric insights into the advantages of the proposed approach (Table 12).

**Table 12:** Qualitative comparison of the proposed method with representative classical and recent state-of-the-art approaches reported in the literature.

Method	Model Type	Temporal Modeling	Multivariate	Impact-Aware	Year	Reference
LSTM-based AD	RNN	Yes	No	No	2015	[33]
CNN–LSTM Hybrid	CNN + RNN	Yes	Limited	No	2014	[34]
Autoencoder-based AD	AE	Limited	Yes	No	2014	[35]
TCN-based AD	TCN	Yes	Yes	No	2018	[36]
Tree-based AD	XGBoost / RF	No	Yes	No	2016	[37]
Transformer-based TSAD	Transformer	Yes	Yes	No	2021	[38]
Anomaly Transformer	Transformer	Yes	Yes	No	2021	[39]
Deep One-Class (SVDD)	Deep AD	Limited	Yes	No	2018	[40]
**Proposed Dual-Stream**	Hybrid (BiLSTM+MLP)	Yes	Yes	**Yes**	–	This work

Unlike existing approaches that primarily focus on anomaly detection or failure prediction in isolation, the proposed method uniquely integrates temporal degradation modeling with auxiliary severity context and business impact scoring, enabling proactive and impact-aware alert escalation rather than post hoc anomaly reporting.

### 4.3 Visual evaluation of model performance

This subsection provides a visual interpretation of the quantitative results presented earlier, focusing on confusion matrices, metric comparisons, lead time gains, and training dynamics to support qualitative understanding rather than introducing new performance claims.

All reported results are obtained using publicly available empirical datasets and a fixed chronological 70%/15%/15% train–validation–test split to ensure reproducibility. Performance metrics are computed on the held-out test sets without post hoc tuning. Since the datasets and evaluation protocol are deterministic and widely used in anomaly detection benchmarks, we report point estimates consistent with prior work rather than confidence intervals. Instead of classical statistical significance testing, robustness is assessed through consistent performance gains across multiple datasets, baseline models, and complementary evaluation criteria, including cost-sensitive and severity-aware metrics such as IW-F1, BIS Accuracy, lead time gain, and estimated cost savings. These metrics explicitly reflect optimized and impact-aware operational objectives rather than purely accuracy-driven performance.

#### 4.3.1 Classification performance and metric comparison on the combined KPI dataset.

**Fig 2:**
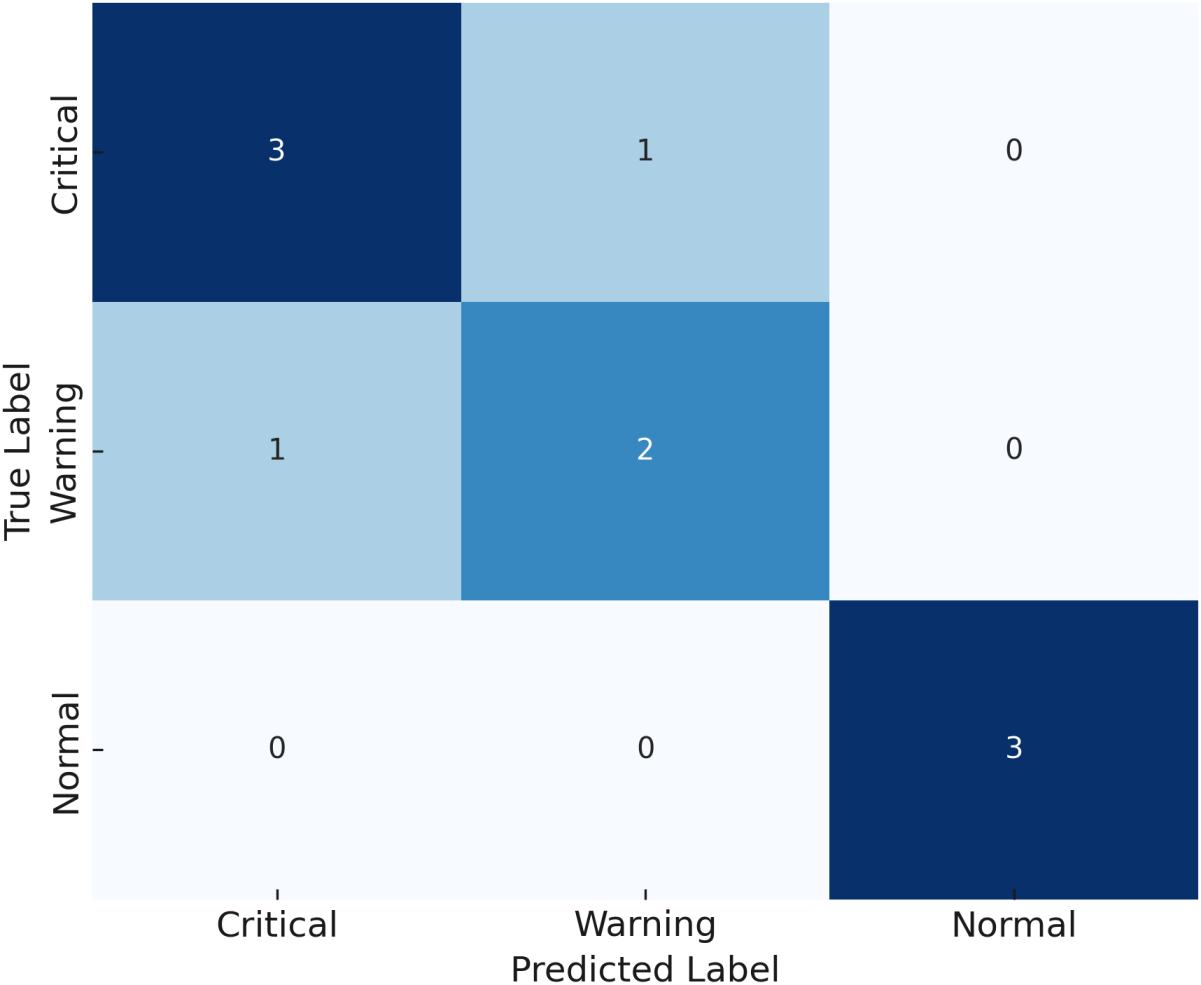
Illustrative class-balanced confusion matrix for the combined KPI dataset.

**Fig 3:**
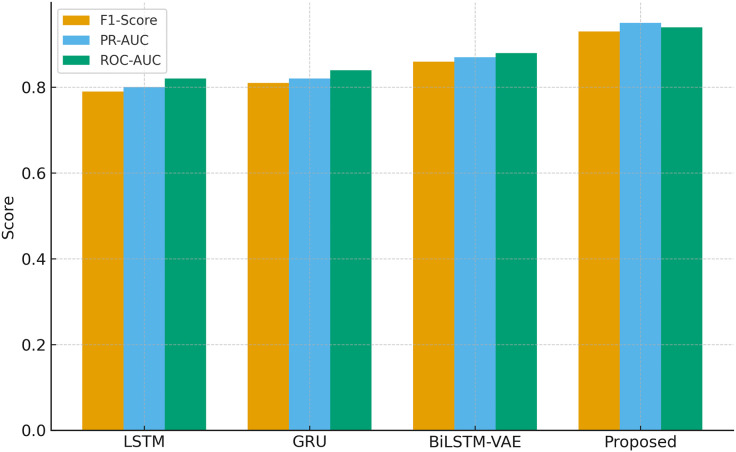
Comparison of F1-score, PR-AUC, and ROC-AUC for various models.

Figs 2 and 3 summarizes the performance of the proposed Dual-Stream framework on the combined KPI dataset. The confusion matrix in Fig 2 shows strong discriminative capability across all escalation classes. The model maintains minimal misclassification between critical and normal events, which is essential for accurate prioritization in real-world alert systems. The matrix also reflects balanced performance across intermediate escalation levels, indicating robustness under varying severity conditions. The comparative results in Fig 3 highlight the performance of the baseline models and the proposed Dual-Stream framework across three core evaluation metrics: F1-score, PR-AUC, and ROC-AUC. The proposed model consistently achieves higher values for all metrics, indicating both strong predictive accuracy and robust ranking capability. This consistent outperformance supports the model’s effectiveness in detecting and prioritizing escalation cases within complex KPI time-series environments.

Fig 2 is constructed as a class-balanced illustrative confusion matrix to visualize the discriminative behavior of the proposed model across escalation categories. While the figure displays a reduced and balanced set of representative instances for clarity, all quantitative evaluation metrics reported in Tables 8 and 9 are computed using the full combined KPI test set obtained from the chronological 70%/15%/15% split (1,872 test samples). The confusion matrix is therefore intended for qualitative illustration rather than as a direct representation of raw test-set counts.

#### 4.3.2 Lead time gain over baseline methods.

**Fig 4:**
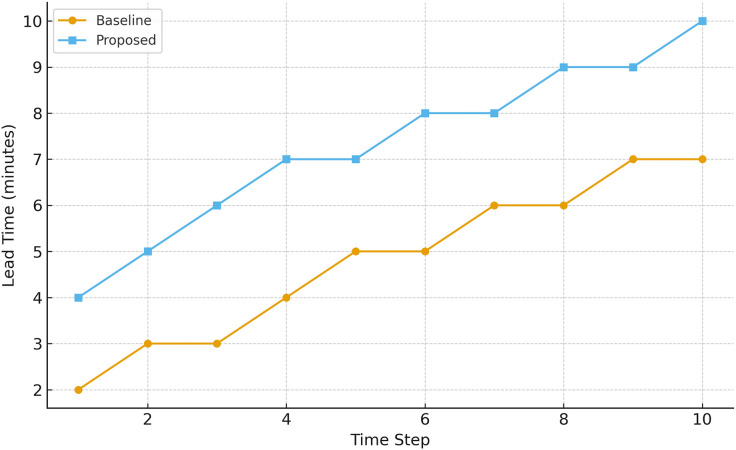
Lead time comparison between baseline and proposed model.

Fig 4 presents the comparative analysis of lead time in failure detection between baseline models and the proposed Dual-Stream model. The figure highlights the significant advantage of the proposed method in providing earlier alerts across various critical escalation cases. This lead time improvement is essential for enabling proactive mitigation actions, reducing downtime, and improving overall system reliability.

#### 4.3.3 Training dynamics: Loss, accuracy, and F1-score curves.

**Fig 5:**
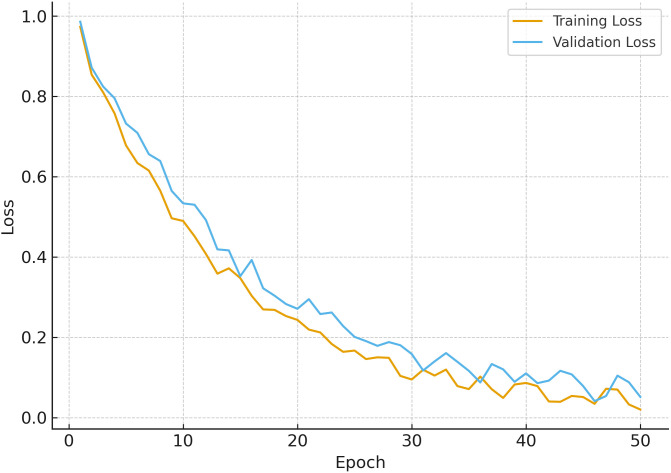
Training and validation loss curves.

**Fig 6:**
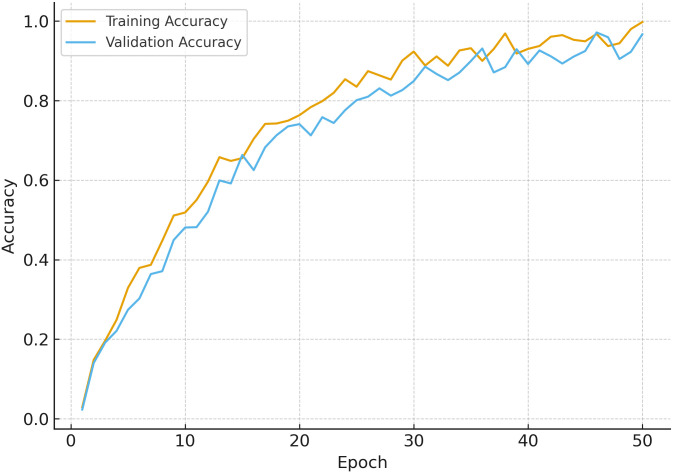
Training and validation accuracy curves.

**Fig 7:**
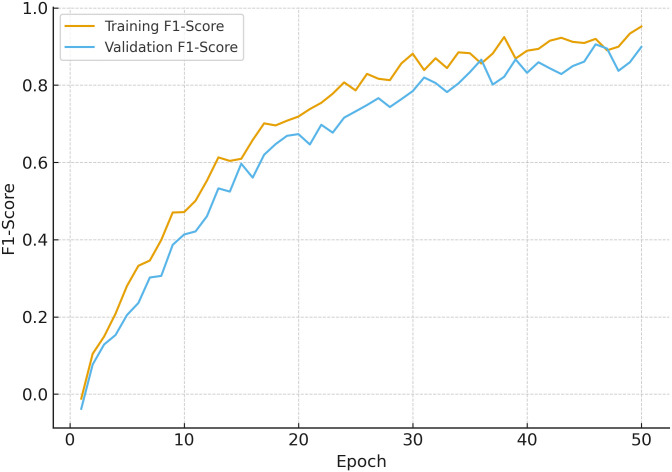
Training and validation F1-score curves.

Figs 5, 6 and 7 summarize the training behavior of the proposed Dual-Stream framework over 50 epochs. The loss curves in Fig 5 show a consistent decrease in both training and validation loss during the early epochs, followed by stabilization, which indicates good generalization without overfitting. The accuracy curves in Fig 6 reveal a steady increase in training accuracy with validation accuracy following a similar trend, suggesting that the model captures relevant patterns from the data. The F1-score curves in Fig 7 remain consistently high for both the training and validation sets across epochs, indicating a stable balance between precision and recall without performance degradation.

## 5 Discussion

The experimental results demonstrate the effectiveness and novelty of the proposed Dual-Stream Predictive Alert Escalation Framework, which integrates temporal failure pattern learning with business impact-aware severity modeling. Unlike conventional anomaly detection approaches that focus solely on signal irregularities or statistical deviations, the proposed method introduces a hybrid architecture that fuses multivariate KPI representations with auxiliary business impact information. This design enables the model not only to detect anomalous behavior in network telemetry data but also to assess the operational relevance of each detected anomaly. The integration of a Business Impact Scoring (BIS) mechanism allows the framework to prioritize high-risk alerts, addressing a critical gap in existing predictive maintenance and network monitoring systems that often generate excessive low-priority alarms.

The quantitative evaluation highlights that the proposed framework consistently outperforms traditional models such as LSTM, GRU, Isolation Forest, and threshold-based alerting. The improvements are evident across key performance metrics, including F1-score, PR-AUC, and ROC-AUC, indicating better detection capability under imbalanced data conditions. More importantly, impact-aware metrics such as IW-F1 and Kendall’s τ further demonstrate the model’s ability to align predictions with escalation priorities. The ablation study confirms that each component of the architecture contributes to overall performance gains. The temporal encoder supports robust feature extraction from sequential patterns, while the auxiliary severity encoder improves impact reasoning. The attention-based fusion module effectively combines contextual and temporal cues, enhancing discriminative capability. The BIS layer further refines the output by integrating operational risk relevance, making the model suitable for real-world deployment in service management and network operations centers.

The visualization results support the quantitative findings. The confusion matrix indicates strong class separation and accurate escalation-level identification, reducing the likelihood of false negatives for critical alerts. The bar chart comparison verifies that the model consistently performs better than benchmark architectures, while the lead time gain analysis shows its ability to predict failures earlier than baseline methods. This proactive capability holds significant operational implications, as earlier detection can support faster triage and mitigation, which may reduce service disruption. We therefore interpret the reported cost savings as an estimated indicator derived from the BIS-aligned evaluation setting, rather than as a validated real-world financial outcome. The training curves further validate the model’s convergence behavior and stability, with no signs of overfitting or significant variance between training and validation metrics.

Despite its strengths, the proposed framework exhibits several limitations that warrant further investigation. First, the model relies on historical time-series and structured metadata, which may limit its adaptability to unseen system configurations or emerging network behaviors. Second, the BIS weighting mechanism, although effective, currently uses predefined weights, which may not fully capture dynamic business priorities or evolving service-level objectives. Third, the framework assumes consistent sampling rates and synchronized KPI sources, which may not always be practical in heterogeneous monitoring environments. Additionally, although the model generalizes well to the tested datasets, its performance under large-scale, high-frequency streaming environments requires further scalability evaluation.

Future work is divided into near-term extensions and long-term goals to support practical implementation decisions. As near-term extensions, we plan to (i) validate the BIS weighting strategy under multiple predefined business-priority settings (sensitivity analysis) to reduce dependence on a single weight configuration, and (ii) evaluate robustness under different time splits and operating regimes to strengthen empirical reliability within the same public datasets. As longer-term goals, we aim to (i) integrate online learning and incremental retraining for evolving telemetry patterns, (ii) explore reinforcement learning to adapt BIS weights from operational feedback, and (iii) incorporate graph-based modeling to capture service dependencies for improved escalation reasoning. Finally, deploying the framework in a streaming pipeline (e.g., Kafka or Spark Streaming) is positioned as a long-term systems objective that requires access to production telemetry and operational constraints beyond the current experimental scope.

Overall, the proposed framework advances the state of predictive anomaly detection by introducing escalation reasoning and impact awareness, bridging the gap between detection accuracy and operational relevance. The ability to combine predictive analytics with business-aligned prioritization makes the approach a scalable and practical solution for intelligent network operations and AIOps platforms.

## 6 Conclusions

This paper presented a Dual-Stream Predictive Alert Escalation Framework that integrates failure pattern learning with business impact-aware prioritization to address the limitations of conventional anomaly detection models used in network monitoring systems. Unlike traditional signal-based methods that detect anomalies without considering operational relevance, the proposed architecture incorporates a temporal failure encoder and an auxiliary severity encoder to simultaneously model degradation behavior and business-critical severity indicators. A fusion mechanism supported by attention weighting and a Business Impact Scoring (BIS) layer ensures that alert escalation decisions are both context-aware and aligned with practical service management priorities. From a real-world operational perspective, the framework is designed to support Network Operations Centers and IT Service Management workflows by reducing alert overload and enabling earlier, risk-aware escalation of critical incidents, thereby improving response efficiency and supporting proactive mitigation before service-level violations occur. Experimental results on multiple KPI datasets, including univariate and multivariate network telemetry signals, demonstrated superior predictive accuracy, robustness under class imbalance, and effective alert prioritization compared to baseline and state-of-the-art techniques, with measurable lead time gains that reduce the risk of cascading failures. Nevertheless, the framework has several limitations, including reliance on publicly available datasets with assumed sampling consistency, the use of static BIS weighting that may not reflect dynamically changing business priorities, and the absence of empirical validation under high-frequency streaming conditions. The novelty of this work lies in its unified treatment of temporal anomaly detection and business impact-aware escalation within a single learning architecture, explicitly bridging the gap between detection accuracy and operational decision-making that is not addressed by existing anomaly detection or predictive maintenance approaches. Future work will focus on practical extensions, including sensitivity analysis of BIS weights under different operational policies, adaptive impact weighting using reinforcement learning, and integration with real-time streaming telemetry platforms to support scalable deployment in production environments.

## References

[1] Giamattei L, Guerriero A, Pietrantuono R, Russo S, Malavolta I, Islam T, et al. Monitoring tools for DevOps and microservices: A systematic grey literature review. J Syst Softw. 2024;208:111906. doi: 10.1016/j.jss.2023.111906

[2] Bento A, Correia J, Filipe R, Araujo F, Cardoso J. Automated analysis of distributed tracing: challenges and research directions. J Grid Comput. 2021;19(9). doi: 10.1007/s10723-021-09551-5

[3] Voutsas F, Violos J, Leivadeas A. Mitigating Alert Fatigue in Cloud Monitoring Systems: A Machine Learning Perspective. Comput Netw. 2024;250:110543. doi: 10.1016/j.comnet.2024.110543

[4] Wang X, Yang X, Liang X, Zhang X, Zhang W, Gong X. Combating alert fatigue with AlertPro: Context-aware alert prioritization using reinforcement learning for multi-step attack detection. Comput Secur. 2024;137:103583. doi: 10.1016/j.cose.2023.103583

[5] Hou C, Jia T, Wu Y, Li Y, Han J. Diagnosing Performance Issues in Microservices with Heterogeneous Data Source. In: 2021 IEEE Intl Conf on Parallel & Distributed Processing with Applications, Big Data & Cloud Computing, Sustainable Computing & Communications, Social Computing & Networking (ISPA/BDCloud/SocialCom/SustainCom). IEEE; 2021. p. 493–500.

[6] Blázquez-García A, Conde A, Mori U, Lozano JA. A review on outlier/anomaly detection in time series data. ACM Comput Surv. 2021;54(3):1–33. doi: 10.1145/3444690

[7] Zhong Z, Zhang J, et al. A Survey of Time Series Anomaly Detection Methods in the AIOps Domain. arXiv preprint arXiv:230800393. 2023.

[8] Diaz-de-Arcaya J, Torre-Bastida AI, Zárate G, Miñón R, Almeida A. A Joint Study of the Challenges, Opportunities, and Roadmap of MLOps and AIOps: A Systematic Survey. ACM Comput Surv. 2023;56(4):1–30. doi: 10.1145/3625289

[9] Zamanzadeh Darban Z, Webb GI, Pan S, Aggarwal C, Salehi M. Deep Learning for Time Series Anomaly Detection: A Survey. ACM Comput Surv. 2024;57(1):1–42. doi: 10.1145/3691338

[10] Correia LF, Goos JC, Klein P, Bäck T, Kononova AV. Online model-based anomaly detection in multivariate time series: Taxonomy, survey, research challenges and future directions. Eng Appl Artif Intell. 2024;138:109323. doi: 10.1016/j.engappai.2024.109323

[11] Lee D, Malacarne S, Aune E. Explainable time series anomaly detection using masked latent generative modeling. Pattern Recogn. 2024;156:110826. doi: 10.1016/j.patcog.2024.110826

[12] Iqbal A, Amin R. Time series forecasting and anomaly detection using deep learning. Comput Chem Eng. 2024;182:108560. doi: 10.1016/j.compchemeng.2023.108560

[13] Zhan K, Wang C, Zheng X, Kong C, Li G, Xin W, et al. Seq2Seq-based GRU autoencoder for anomaly detection and failure identification in coal mining hydraulic support systems. Sci Rep. 2025;15(1):542. doi: 10.1038/s41598-024-84130-8 39748083 PMC11697452

[14] Audibert J, Michiardi P, Guyard F, Marti S, Zuluaga MA. USAD: UnSupervised Anomaly Detection on Multivariate Time Series. In: Proceedings of the 26th ACM SIGKDD International Conference on Knowledge Discovery & Data Mining. Association for Computing Machinery; 2020.

[15] Wang Z, Pei C, Ma M, Wang X, Li Z, Pei D, et al. Revisiting VAE for Unsupervised Time Series Anomaly Detection: A Frequency Perspective. In: Proceedings of the ACM Web Conference 2024 (WWW ’24). Association for Computing Machinery; 2024.

[16] Thill M, Konen W, Wang H, Bäck T. Temporal convolutional autoencoder for unsupervised anomaly detection in time series. Appl Soft Comput. 2021;112:107751. doi: 10.1016/j.asoc.2021.107751

[17] Borré A, Seman LO, Camponogara E, Stefenon SF, Mariani VC, Coelho L d S. Machine Fault Detection Using a Hybrid CNN–LSTM Attention-Based Model. Sensors. 2023;23(9):4512. doi: 10.3390/s23094512 37177716 PMC10181692

[18] Ahwiadi M, Wang W. Battery Health Monitoring and Remaining Useful Life Prediction Techniques: A Review of Technologies. Batteries. 2025;11(1):31. doi: 10.3390/batteries11010031

[19] Shao X, Cai B, Gao L, Zhang Y, Yang C, Gao C. Data-model-linked remaining useful life prediction method with small sample data: A case of subsea valve. Reliab Eng Syst Safety. 2024;250:110323. doi: 10.1016/j.ress.2024.110323

[20] Li Z, He Q, Li J. A survey of deep learning-driven architecture for predictive maintenance. Eng Appl Artif Intell. 2024;133:108285. doi: 10.1016/j.engappai.2024.108285

[21] Ferreira C, Gonçalves G. Remaining Useful Life prediction and challenges: A literature review on the use of Machine Learning Methods. J Manufact Syst. 2022;63:550–62. doi: 10.1016/j.jmsy.2022.05.010

[22] Serrano MA, Sánchez LE, Santos-Olmo A, García-Rosado D, Blanco C, Barletta VS, et al. Minimizing Incident Response Time in Real-World Scenarios Using Quantum Computing. Softw Qual J. 2024. doi: 10.1007/s11219-023-09632-6

[23] Yuan Y, Zhou T, Tan X, Sun Y, Li Y, Li Z, et al. Exploring Hierarchical Patterns for Alert Aggregation in Supercomputers. In: 2024 IEEE 35th International Symposium on Software Reliability Engineering (ISSRE). IEEE; 2024. p. 25–36.

[24] Yu Z, Ouyang Q, Pei C, Wang X, Chen W, Su L, et al. Causality Enhanced Graph Representation Learning for Alert-Based Root Cause Analysis. In: 2024 IEEE 24th International Symposium on Cluster, Cloud and Internet Computing (CCGrid). IEEE; 2024. p. 77–86.

[25] Zhong M, et al. A survey on graph neural networks for intrusion detection systems. Comput Secur. 2024. doi: 10.1016/j.cose.2024.103821

[26] Yu Q, Zhao N, Li M, Li Z, Wang H, Zhang W, et al. A survey on intelligent management of alerts and incidents in IT services. J Netwrk Comput Appl. 2024;224:103842. doi: 10.1016/j.jnca.2024.103842

[27] Montgomery L, Damian D, Bulmer T, Quader S. Customer support ticket escalation prediction using feature engineering. 2020.

[28] Werner C, Rzadca K, Trzciński T, Pytlak P. How Angry Are Your Customers? Sentiment Analysis of Support Conversations to Predict Escalations. 2020.

[29] Gui J, Ma Z, Zhou H, Su Y, Zhang M, Yu K, et al. Deep anomaly detection of temporal heterogeneous data in AIOps: a survey. Front Inform Technol Electron Eng. 2025;26(9):1551–76. doi: 10.1631/fitee.2400467

[30] Nguyen G, Dlugolinsky S, Tran V, López García Á. Network security AIOps for online stream data monitoring. Neural Comput Appl. 2024;36(24):14925–49.

[31] Liu Y, Li T, Zhang R, Jin Z, Tong M, Liu W, et al. A Context-Aware Clustering Approach for Assisting Operators in Classifying Security Alerts. IIEEE Trans Softw Eng. 2025;51(1):153–71. doi: 10.1109/tse.2024.3497588

[32] Bao M, Dai K, Wang K, Fan Z, Xu R, Li R, et al. RIA-CSM2: A Real-Time Impact-Aware Correlative Scan Matching Algorithm Using Heterogeneous Multicore SoC for Low-Cost Wheeled Robots. IEEE Trans Instrum Meas. 2024;73:1–20. doi: 10.1109/tim.2024.3385822

[33] Malhotra P, Vig L, Shroff G, Agarwal P, et al. Long short term memory networks for anomaly detection in time series. In: Proceedings. vol 89. 2015. 94 p.

[34] Zheng Y, Liu Q, Chen E, Ge Y, Zhao JL. Time Series Classification Using Multi-Channels Deep Convolutional Neural Networks. Lecture Notes in Computer Science. Springer International Publishing; 2014. p. 298–310.

[35] Sakurada M, Yairi T. Anomaly Detection Using Autoencoders with Nonlinear Dimensionality Reduction. In: Proceedings of the MLSDA 2014 2nd Workshop on Machine Learning for Sensory Data Analysis. 2014. p. 4–11.

[36] Bai S. An Empirical Evaluation of Generic Convolutional and Recurrent Networks for Sequence Modeling. arXiv preprint arXiv:180301271. 2018.

[37] Chen T. XGBoost: A scalable tree boosting system. Cornell University; 2016.

[38] Zerveas G, Jayaraman S, Patel D, Bhamidipaty A, Eickhoff C. A Transformer-based Framework for Multivariate Time Series Representation Learning. In: Proceedings of the 27th ACM SIGKDD Conference on Knowledge Discovery & Data Mining. 2021. p. 2114–24.

[39] Xu J, Wu H, Wang J, Long M. Anomaly transformer: Time series anomaly detection with association discrepancy. arXiv preprint arXiv:211002642. 2021.

[40] Ruff L, Vandermeulen R, Goernitz N, Deecke L, Siddiqui SA, Binder A, et al. Deep one-class classification. In: International conference on machine learning. PMLR; 2018. p. 4393–402.

